# Construction of m7G subtype classification on heterogeneity of sepsis

**DOI:** 10.3389/fgene.2022.1021770

**Published:** 2022-11-24

**Authors:** Jinru Gong, Jiasheng Yang, Yaowei He, Xiaoxuan Chen, Guangyu Yang, Ruilin Sun

**Affiliations:** ^1^ Department of Pulmonary and Critical Care Medicine, Guangdong Second Provincial General Hospital, Guangzhou, China; ^2^ The Second School of Clinical Medicine, Southern Medical University, Guangzhou, China

**Keywords:** sepsis, N7-methylguanosine, heterogeneity, prognosis, integrated analysis

## Abstract

Sepsis is a highly heterogeneous disease and a major factor in increasing mortality from infection. N7-Methylguanosine (m7G) is a widely RNA modification in eukaryotes, which involved in regulation of different biological processes. Researchers have found that m7G methylation contributes to a variety of human diseases, but its research in sepsis is still limited. Here, we aim to establish the molecular classification of m7G gene-related sepsis, reveal its heterogeneity and explore the underlying mechanism. We first identified eight m7G related prognostic genes, and identified two different molecular subtypes of sepsis through Consensus Clustering. Among them, the prognosis of C2 subtype is worse than that of C1 subtype. The signal pathways enriched by the two subtypes were analyzed by ssGSEA, and the results showed that the amino acid metabolism activity of C2 subtype was more active than that of C1 subtype. In addition, the difference of immune microenvironment among different subtypes was explored through CIBERSORT algorithm, and the results showed that the contents of macrophages M0 and NK cells activated were significantly increased in C2 subtype, while the content of NK cells resting decreased significantly in C2 subtype. We further explored the relationship between immune regulatory genes and inflammation related genes between C2 subtype and C1 subtype, and found that C2 subtype showed higher expression of immune regulatory genes and inflammation related genes. Finally, we screened the key genes in sepsis by WGCNA analysis, namely NUDT4 and PARN, and verified their expression patterns in sepsis in the datasets GSE131761 and GSE65682. The RT-PCR test further confirmed the increased expression of NUDTA4 in sepsis patients. In conclusion, sepsis clustering based on eight m7G-related genes can well distinguish the heterogeneity of sepsis patients and help guide the personalized treatment of sepsis patients.

## Introduction

Sepsis is a highly heterogeneous disease characterized by life-threatening organ dysfunction caused by dysregulation of host response to infection, which is the main factor to increase infection mortality (Singer et al., 2016). Globally, sepsis remains a serious public health issue. Sepsis affects about 20 million cases per year, and the mortality rate is about 26% (Fleischmann et al., 2016). The treatment of sepsis mainly includes the combination of early antibiotics, fluid resuscitation and symptomatic treatment of vasopressors, which can significantly improve the symptoms of patients. The risk of infection, however, increases with long-term hospitalization and invasive operation. Conversely, frequent drug treatment can easily lead to multiple drug-resistant bacterial infections, leading to multiple organ failure and other symptoms, which posessevere challenges to the treatment and management of sepsis (Zhang and Ning, 2021). In addition, due to the high heterogeneity of sepsis, there is no proven standard or disease classification that can effectively guide the treatment of patients (Purcarea and Sovaila, 2020). Therefore, it is essentialto explore the molecular mechanism of disease progression and the classification of related diseases for the treatment of sepsis.

RNA methylation regulates gene expression at the post transcriptional level and is an epigenetic regulatory mode. At present, more than 150 RNA methylation modifications have been found in eukaryotes. Among them, m7G methylation modification is one of the most common base modifications in post transcriptional regulation, which widely occurs in the 5’ cap region of tRNA, rRNA and eukaryotic mRNA (Liu et al., 2017; Roundtree et al., 2017). m7G modification is involved in the regulation of various processes, such as mRNA transcription, splicing and translation. More and more studies have found that the occurrence of many human diseases is related to the methylation modification of m7G. Bing et al. found that there were significant differences in m7G mRNA modification in drug-resistant AML cells, and the low methylated m7G modification level was significantly enriched in ABC transporter related mRNA, suggesting that the down-regulation of m7G methylation can actively regulate ABC transporter related genes in AML cells, leading to drug resistance in AML (Zhang et al., 2022). Chen et al., 2022 confirmed that m7G methyltransferase METTL1promotes the development of HNSCC by regulating PI3K/Akt signaling pathway, and changes the immune microenvironment and intercellular communication between HNSCC tumors and stroma. Epigenetic regulation plays a central role in the pathogenesis of sepsis (Binnie et al., 2020), but the role and mechanism of m7G in sepsis remain unclear.

In order to solve the above problems, this study intends to explore the role and potential molecular mechanism of m7G related genes in sepsis, classify sepsis patients into different subtypes according to m7G-related genes. The pathway enrichment and immune microenvironment were explored to explain the potential mechanism of heterogeneity of different subtypes, and the key regulatory genes NUDT4 and PARN were identified, which are significantly correlated with the prognosis of septic shock patients. This study provides the basis for the treatment and management of septic patients.

## Materials and methods

### Data acquired

GEO database (Barrett et al., 2013) (https://www.ncbi.nlm.nih.gov/) is a gene expression database maintained by NCBI, which stores gene expression data uploaded by research institutions around the world. In this study, GSE65682 dataset was downloaded from GEO database, including 42 healthy control samples and 760 sepsis samples. We extracted 479 samples with complete survival data for classification in sepsis patients. We downloaded GSE131761 dataset from GEO database, including 15 healthy control samples and 81 septic shock samples, to further explore the expression of m7G related regulatory genes. m7G gene was obtained through previous literature.

### Prognostic genes screening and consensus clustering

In order to further classify the prognosis of sepsis patients, we selected m7G-related genes for consensus clustering (Swift et al., 2004). The selection of prognostic genes was based on Kaplan Meier (KM) survival analysis (Goel et al., 2010) (*p* < 0.05). After screening the prognostic m7G-related genes, the consensus clustering was used for subtype clustering. About 80% of the samples were analyzed in each iteration, and a total of 50 iterations were performed. The optimal cluster number is determined by the cumulative distribution function (CDF) curve of the consistency score, the clear difference between groups in the consistency matrix heatmap, and the characteristics of the consistency cumulative distribution function map. KM curve will be used to evaluate the prognosis of different m7G sepsis subtypes with a cutoff value of *p* < 0.05.

### WGCNA network construction

WGCNA (Langfelder and Horvath, 2008) is a method to summarize gene expression data into different coexpression modules, which can be used to explore the relationship between different modules and the correlation between modules and clinical symptoms. We taked the genes with the highest variance of 5,000 within the gene expression data to construct the coexpression network. *β* is a soft threshold, which is related to the independence and average connectivity of modules. The soft threshold of this study is setting as 12. The topological overlap degree matrix (TOM) represents the overlap of network neighbors. The hierarchical clustering method is used to construct the cluster tree structure of TOMmatrix, and the dynamic tree cutting method of pheatmap package is used to cluster graphs. Based on the subtype information of different m7G-related sepsis, the correlation between different modules and clinical phenotype was evaluated. The relationship between the module and the clinical phenotype was calculated by Pearson correlation test. *p* < 0.05 was defined as significant correlation. The module with the largest correlation was selected for subsequent analysis.

### Go and KEGG functional enrichment

ssGSEA (Barbie et al., 2009) is an extension of the GSEA method, which allows the definition of an enrichment score that represents the absolute enrichment of gene sets in each sample within a given dataset. In this study, the ssGSEA algorithm of GSVA package was used to evaluate the pathway levels of GO and KEGG in different subtypes. The GO items included three categories: biological process (BP), molecular function (MF) and cellular component (CC). The background gene set used for the analysis was from the GSVA database (Liberzon et al., 2015) (C2 and C5).

### Immune gene correlation analysis

CIBERSORT (Chen et al., 2018) deconvolution algorithm can estimate the composition and relative abundance of immune cells in mixed cells based on gene transcriptome data. In this study, CIBERSORT LM22 was used to estimate the expression matrix of immune cell characteristics, and then the CIBERSORT algorithm was used to quantify the relative proportion of immune cell infiltration in different sepsis subtypes, and to compare the differences of immune cells between these two groups. The correlation analysis between the key genes and the content of immune cells is carried out to evaluate the relationship between the key genes and immune cells. It is considered statistically significant if *p* is less than 0.05.

### RT-PCR validation of hub genes

A total of 10 participants were recruited from the Department of Pulmonary and Critical Care Medicine of the Guangdong Second Provincial General Hospital, including 5 sepsis cases and 5 non-septic patients. The study was carried out in accordance with the Helsinki Declaration and was approved by the Ethics Committee of the Second People’s Hospital of Guangdong Province. The whole blood samples of each case were collected into tubes with EDTA. Total RNA was isolated using the TRIzol reagent (TIANGEN, CHINA) in line with the manufacturer’s instructions. The real-time PCR was conducted using AOPR-1200 detection kit (Genecopies, China). The sequences of primers for the indicated genes were as follows: GAPDH forward (F), CAA​GAG​CAC​AAG​AGG​AAG​AGA​G and reverse (R), CTA​CAT​GGC​AAC​TGT​GAG​GAG; NUDT4 F, CCT​CCT​AAA​GTG​CTG​GGA​TTA​C and R, CAA​AGT​CCT​GGG​AGA​GAA​GAA​A; PARN F, CAA​AGT​GTA​CCA​GGC​CAT​AGA​G, and R, CTG​AAG​GTC​CAT​CAC​TGA​TTC​C.

### Statistic method

R program (4.1.2) was used to analyze data and dram diagrams. The differences between subgroups were tested by Wilcox test, and all correlations were calculated by pearson method. Survival curves were generated by Kaplan-Meier method and compared by log rank. *p* < 0.05 with statistical significance.

## Result

### Prognosis of m7G related genes in sepsis

**FIGURE 1:**
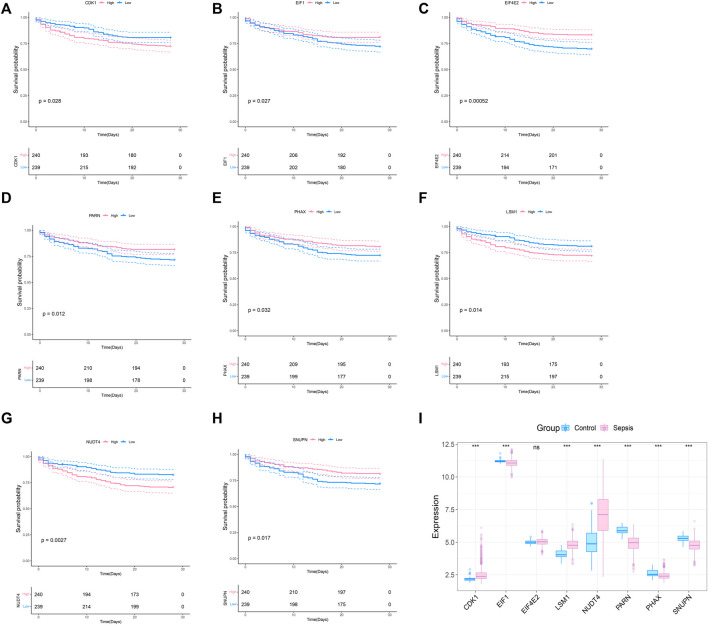
Screening of m7G-related prognosis genes. **(A–H)** Survival analysis of m7G gene related to sepsis prognosis. The pink indicates high expression and blue indicates low expression. **(I)** Expression profile of 8 prognostic genes in sepsis, and the majority of genes are dysregulated in sepsis. The blue indicates control samples and pink indicates sepsis samples.

To explore whether the molecular classification of the m7G related genes in sepsis can explain the heterogeneity of sepsis patients, we acquired the expression data of GSE65682 from the GEO database, with a total of 802 samples, including 42 healthy control samples and 760 sepsis samples. We extracted 479 samples with complete survival status for follow-up analysis. 42 m7G related genes were obtained from the previous literature, and then the list was limited to the genes with available RNA expression data in GSE65682, leaving 33 m7G regulated genes. They are EIF4E2, NUDT4, PARN, LSM1, SNUPN, EIF1, CDK1, PHAX, CYFIP1, CCNB1, NCBP1, EIF4E3, DCPS, NSUN2, EIF4A1, JUND, LARP1, NUDT3, APAF1, EIF3D, XPO1, WDR4, NCBP2, GEMIN5, EIF4E, IFIT5, NUDT16, IPO8, METTL1, DCP2, TGS1, EIF4G1, and EIF4G3. We performed survival analysis on these 33 genes and screened the prognosis-related m7G genes. The results showed that a total of 8 prognosis related genes were screened (*p* < 0.05) (Figures 1A–G). In addition, we analyzed the expression of these 8 m7G genes in normal samples and sepsis samples, and the results showed that 7 genes showed different expression in two groups of samples (Figure 1I).

### Molecular classification of prognosis-related m7G genes in sepsis

**FIGURE 2:**
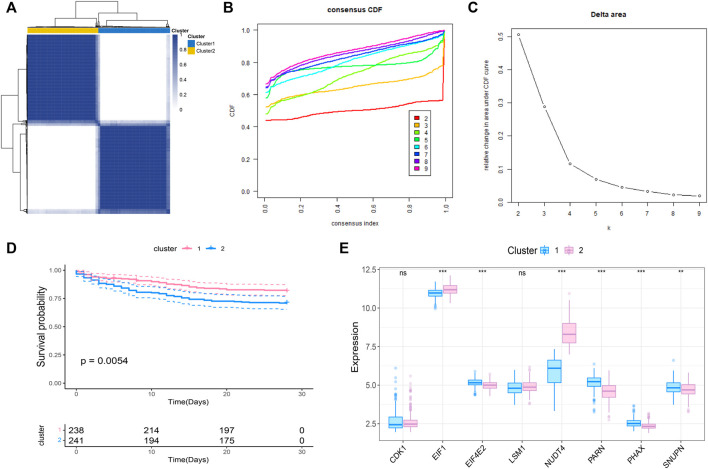
Molecular classification of sepsis based on m7G-related prognosis genes. **(A)** Consensus clustering heatmap: when *k* = 2, the clustering of samples is relatively stable. **(B)** The cumulative distribution function (CDF) curve. **(C)** The delta area score displays the survival curves of these two subtypes (*k* = 2) with the relative increase of cluster stability **(D)**. Kaplan-Meier curve showed the statistical difference between these two subtypes (*p* < 0.0001), and the prognosis of patients within Cluster 2 was poor. **(E)** Expression profile of m7G gene in the two subtypes.

We further conducted the consensus clustering and performed molecular classfication of GSE65682 based on the expression of the m7G related prognostic genes. The results showed that the boundary between the two subtypes of the sample was clear when *k* = 2, so the sepsis was divided into two clusters (Figures 2A–C). In addition, KM survival analysis showed that the survival of these two clusters was significantly different (Figure 2D), suggesting that there were different survival outcomes between the two subgroups. Therefore, it is particularly important to further explore the molecular characteristics of the two subgroups. We also analyzed the expression differences of 8 genes in the two subtypes, and the results are shown in the figure (Figure 2E).

### Functional enrichment among subtypes

**FIGURE 3:**
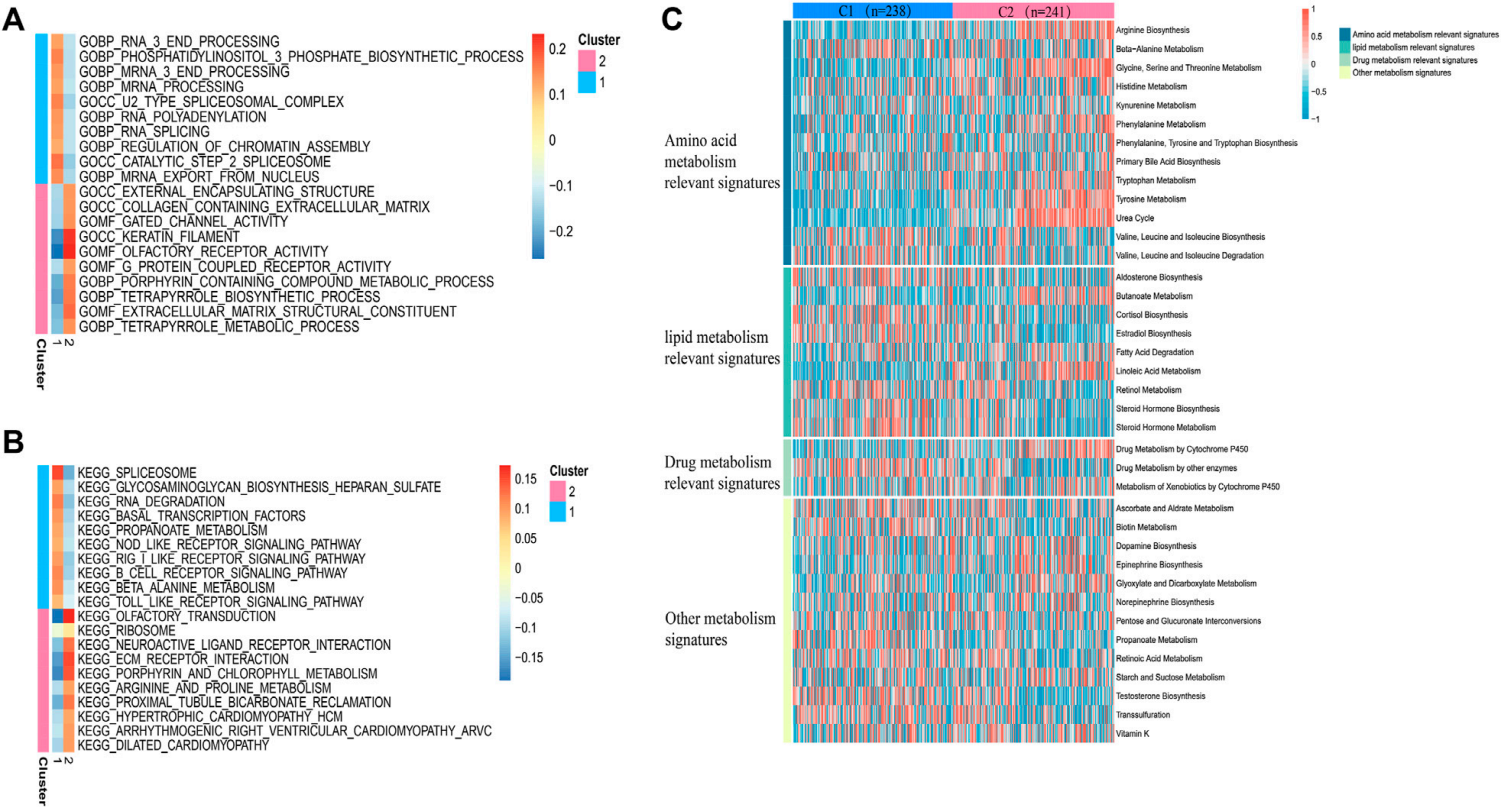
Pathway analysis of sepsis pathogenesis. **(A,B)** 20 pathways with significant enrichment of GO/KEGG in the two subtypes. **(C)** Heatmaps of specific metabolic-related pathways.

In view of the different survival outcomes between C1 subtype and C2 subtype, this study will use ssGSEA to explore the molecular mechanism of different subtypes and clarify the reasons for the different survival outcomes of these patients. We selected the 20 most representative pathways for cluster 1 and cluster 2 to make the visualization, which revealed different pathways enriched in each subtype (Figures 3A,B). The results of GO analysis showed that cluster 2 was significantly related to the pathway of porphyrin containing combined metabolic process, tetrapyrole biological process extra cellular matrix structural construct, tetrapyrole metabolic process. KEGG analysis showed that cluster 2 was significantly correlated with ECM receptor interaction, porphyrin and chlorophyll metabolism, and arginine and proline metabolism pathways. The above results suggest that the subtype differences may be closely related to the metabolic activities. We further quantified four different metabolic activities, and the results suggest that the amino acid metabolic activities in patients with C2 subtype are more active than those of C1 subtype (Figure 3C).

### Heterogeneity of immune status among m7G subtypes

**FIGURE 4:**
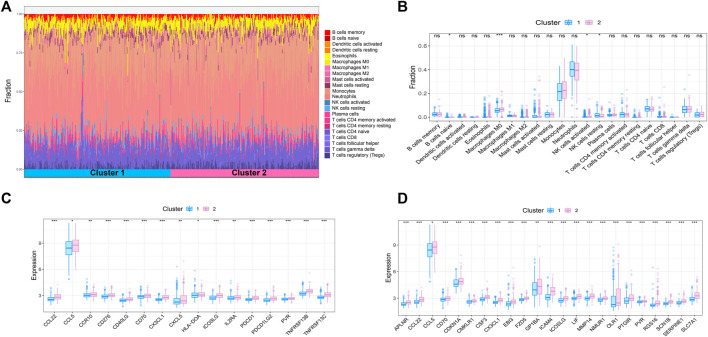
Immune microenvironment analysis of sepsis. **(A)** The percentage of each immune cell content within the sample **(B)** The difference of immune cell content between subtypes, *p* < 0.05 was considered statistically significant. **(C,D)** Expression patterns of immune and inflammatory factors in sepsis.

Sepsis is an acute organ dysfunction syndrome caused by physiological and pathophysiological responses to infection. Immune dysfunction is related to abnormal coagulation, endothelial and epithelial barrier destruction, and leads to changes in vascular activity of multiple organ dysfunction. Therefore, this study intends to further explore the difference of immune microenvironment among different subtypes through CIBERSORT algorithm. The results showed that compared with cluster 1, the contents of macrophages M0 and NK cells activated in cluster 2 were significantly increased, while the content of NK cells resting was significantly decreased in cluster 2 (Figures 4A,B). In addition, some immune related genes and inflammation related genes were obtained from TISIDB database and GSEA database to verify the expression of immune genes and inflammation genes in the two subtypes, respectively. The results showed that the genes were up-regulated in cluster 2 (Figures 4C,D).

### Construction of WGCNA network based on molecular classification and RT-PCR validation of hub genes

**FIGURE 5:**
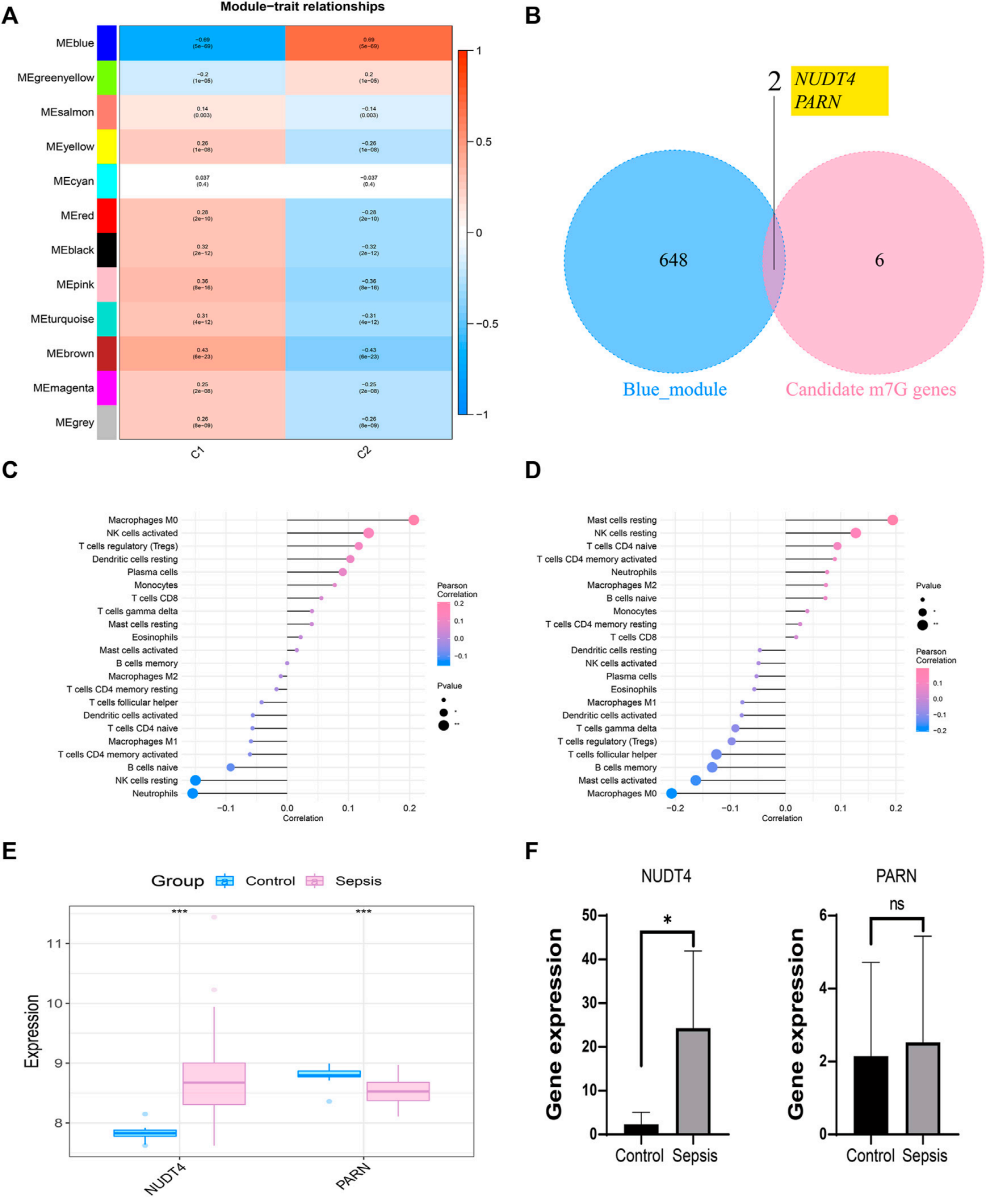
Construction of weighted co-expression network and identification of key genes. **(A)** The heatmap of the correlation between the module characteristic genes and the clinical of sepsis subtypes. The blue module with the highest correlation was selected for subsequent analysis. **(B)** Identification of key genes in sepsis. **(C)** Correlation between NUDT4 and immune cells. **(D)** Correlation between PARN and immune cells. **(E)**Verification of the expression of key genes in external validation dataset. **(F)** The RT-PCR validation of key genes expression.

We took the classification status of two different sepsis subpopulations as clinical symptoms, further constructed WGCNA network according to the expression profile data of GSE65682, screened the key genes affecting sepsis progression, and explored the sepsis related gene regulatory network. The soft threshold of WGCNA network was set to 12, and 12 gene modules were co classified. It was found that the blue module had the highest correlation with sepsis subtypes [cor = 0.69, *p*= (5e − 69)] (Figure 5A). We intersected 650 genes of the blue module with 8 prognosis related m7G genes to obtain 2 intersection genes, which are NUDT4 and PARN respectively (Figure 5B). The two genes are strongly correlated with the content of immune cells, among which NUDT4 is positively correlated with Macrophages M0, NK cells activated, T cells regulatory (Tregs), Dendritic cells resting, Plasma cells, and negatively correlated with Neutrophils, NK cells resting, and B cells naive (Figure 5C). PARN is positively correlated with Mast cells resting, NK cells resting, T cells CD4 naive, and negatively correlated with macrophages M0, Mast cells activated, *etc.* (Figure 5D). We also explored the expression of two key genes through the GSE131761 and GSE65682 datasets, and the results showed that the expression trends of NUDT4 and PARN were consistent in the two datasets (Figure 5E). Additionally, we collected samples from sepsis patients and control cases to explore the expression pattern of hub genes. The results showed that the expression of NUDT4 was significantly increased in sepsis patients (Figure 5F).

### Correlation between key genes and disease regulating genes

**FIGURE 6:**
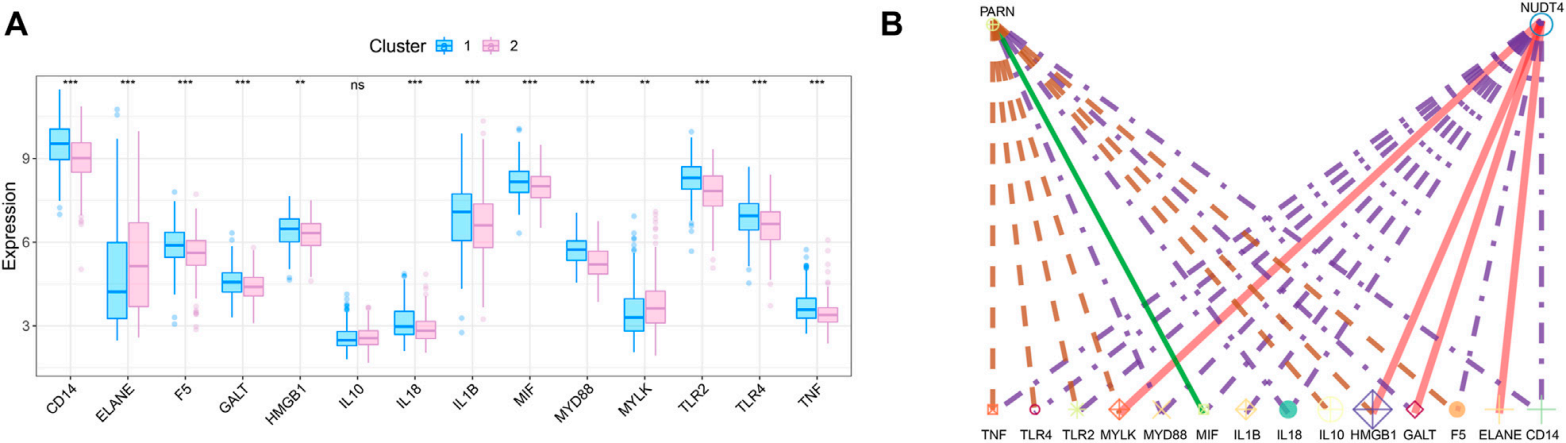
Correlation analysis of m7G-related genes and sepsis gene. **(A)** The expression difference of sepsis-related genes between the two subtypes. **(B)** Regulatory network of key genes and sepsis-related genes.

We obtained the disease regulatory genes of sepsis according to GENECARD database, and selected the top 20 genes of relevance for difference analysis (Figure 6A). In order to explore the relationship between key genes and sepsis disease regulation, we performed correlation analysis on key genes and 20 disease regulation genes. The regulatory network of m7G regulatory genes and sepsis related genes is shown in Figure 6B.

## Discussion

Sepsis is a disease of multiple organ dysfunction caused by the rapid response of the host to infection (Huang et al., 2019). Worldwide, sepsis affects about 30 million people every year and is one of the main causes of death of critically ill patients. In hospitalized patients, any infection may lead to sepsis, and its incidence rate is about 1%–2% (Huang et al., 2019). Therefore, its treatment cost is also the highest (Rocheteau et al., 2015). In recent years, sepsis related research has been developing, but its incidence rate and mortality are still increasing. Sepsis patients are also facing many adverse effects such as physical and psychological (Iwashyna et al., 2012; Gaieski et al., 2013). In addition, due to the different clinical manifestations of sepsis and the obvious heterogeneity among patients (Huang et al., 2019), there are still many challenges in the diagnosis, treatment and management of sepsis.

Epigenetic regulation plays a key role in the occurrence and development of sepsis. Epigenetic changes mainly refer to the changes in gene expression caused by the body in response to external stimuli, which are the payment of environmental factors and genetic factors. After the patients with sepsis are stimulated by external infectious factors, the body will have an inflammatory storm. The host pathogen interaction will lead to the epigenetic changes of the host key inflammatory regulatory genes (Binnie et al., 2020). In some animal models, treatment with epigenetic modifiers can alleviate sepsis related organ damage and improve its survival (Cao et al., 2014; Shih et al., 2016). m7G is one of epigenetic regulation. It is known that m7G regulation plays an important role in a variety of human diseases (Malbec et al., 2019; Luo et al., 2022), but its role in septic shock is still unclear. We previously analyzed the potential role of m7G in septic shock through the GEO dataset and found that 8 m7G related genes were closely related to the prognosis of septic shock patients. Based on these 8 m7G related genes, two different subtypes (C1 and C2) were identified by consensus clustering, and it was found that patients with C2 subtype had poor prognosis. In addition, the results of pathway analysis showed that the amino acid metabolism of C2 subtype was more active than that of C1 subtype. The results of immune infiltration analysis showed that the contents of macrophages M0 and NK cells activated in C2 subtype were significantly increased, while the content of NK cells resting decreased significantly in C2 subtype. Additionally, C2 patients had higher expression levels of immune related genes and inflammation related genes. Finally, we screened out two genes that were significantly associated with sepsis prognosis, NUDT4 and PARN.

Systemic inflammatory response syndrome sepsis syndrome is associated with hypermetabolism, increased oxygen consumption and energy consumption, activation of peripheral protein catabolism, especially enhanced metabolic activity in the liver and viscera (Dahn et al., 1987; Barton and Cerra, 1989). Under physiological conditions, the liver mainly synthesizes constituent proteins, such as albumin and transferrin, while in sepsis, the synthesis changes from constituent proteins to acute phase proteins, including procalcitonin, C-reactive protein, complement factor, binding globin *α* 2-macroglobulin and *α* 1-acid glycoprotein (Lang et al., 2007; Remick, 2007). In addition, in sepsis, citrulline synthesis is reduced, but the decomposition is increased, and the progressive decrease of citrulline content in the body is one of the reasons for the suppression of macrophage function. Citrulline supplementation can improve the synthesis of endogenous arginine and NO, thus improving the prognosis of sepsis (Xiao et al., 2015). Studies have shown that the level of branched chain amino acids is related to the severity of sepsis, and can predict the risk of death of sepsis patients in ICU (Reisinger et al., 2021). Our study also suggests that the amino acid metabolism level of C2 subtype patients is significantly higher than that of C1 subtype. The poor prognosis of C2 subtype patients may be related to the amino acid metabolism level *in vivo*. On the other hand, changes in lipid metabolism during sepsis are protective reactions against infection, and changes in lipid mass spectrometry are directly related to inflammation (Bermudes et al., 2018). Lipoproteins have the ability to bind and neutralize toxic bacterial substances that regulate cytokine production during inflammation, thereby weakening host response (Murch et al., 2007). Our results also revealed changes in lipid metabolism pathways between different subtypes.

Sepsis is an acute organ dysfunction syndrome caused by physiological and pathophysiological responses to infection. Immune dysfunction may trigger coagulation abnormalities, endothelial and epithelial barrier disruption, and ultimately vascular activity changes leading to multiple organ dysfunction (Kotas and Matthay, 2018). However, the specific cellular and molecular pathways responsible are not fully understood. Our study showed that the M0 level of C2 subtype macrophages was significantly higher than that of C1 subtypes. Macrophages, as the first line of defense against pathogens, are rapidly activated by alveolar macrophages in the early stage to fight against infection and promote the regression of inflammation in the later stage (Hussell and Bell, 2014). Among them, IFN-r can enhance the release of IL-1, IL-6, IL-8, and tumor necrosis factor (TNF), IL-4, IL-13, and IL-10 from monocytes/macrophages exposed to LPS, and inhibit the production of pro-inflammatory factors. And a large number of pro-inflammatory and anti-inflammatory production are related to the occurrence of sepsis (Cavaillon and Adib-Conquy, 2005). In addition, the balance between lung tissue cells and peripheral blood mononuclear cell-derived macrophages may be an important marker of inflammatory balance during lung infection. In patients with severe coronavirus infection, peripheral monocytes/macrophages increased significantly while alveolar macrophages decreased significantly, suggesting that the abnormal number of peripheral monocytes/macrophages and alveolar macrophages may also be an important cause of immune imbalance (Liao et al., 2020). Therefore, in addition to focusing on the production of cytokines by macrophages to participate in the pathogenesis of sepsis, macrophages in different positions also need to be focused. Interestingly, recent studies have shown that M0 macrophages do not conform to standard M1 or M2 models, and they are more similar to M2 macrophages, which may represent another type of TAM. M0 macrophages are one of the cell subsets closely related to the poor prognosis of breast cancer (Ali et al., 2016), prostate cancer (Jairath et al., 2020) and lung adenocarcinoma (Liu et al., 2017). Our results also suggest that M0 macrophages are significantly increased in C2 subtypes with poor prognosis in sepsis. On the other hand, NK cell resting was also significantly different between C1 and C2 subtypes. NK cells are abundant in tissues such as lung, liver, spleen and blood (Grégoire et al., 2007). IFN-γ, GM-CSF and TNF-α are major cytokines produced by activated NK cells (Huntington et al., 2007), and have a protective effect during infection but a deleterious effect during aseptic or infectious systemic inflammatory response syndrome. In addition, studies have also shown that NK cell function can be affected by IL-10 and TGF- β-1 (Scott et al., 2006; Ralainirina et al., 2007).

Our study also identified the key genes that significantly affect the prognosis in sepsis, namely NUDT and PRAN. NUDTS catalyze the hydrolysis of various nucleoside pyrophosphates associated with other amino acid moieties (Bessman, 2019). In the process of eliminating hydrolytic substrates, NUDT plays a signaling and regulatory role in metabolism (Mildvan et al., 2005). Studies have shown that NUDT4 can be used as a prognostic target related to m7G methylation in gastric cancer, and the prognosis model based on this can better predict the prognosis of gastric cancer patients (Li et al., 2022). However, there is a lack of relevant research on NUDT4 in infectious diseases and even sepsis. PARN, a major mammalian deadenylase, is the only known enzyme that binds both the 5′cap structure and the 3′ poly A, thereby increasing the degradation rate and enhancing its sustained synthesis ability. PARN is important in oocyte maturation, embryogenesis, early development, DNA damage and cell cycle progression. This enzyme is also involved in the regulation of nonsense mediated mRNA decay and cytoplasmic polyadenylation (Balatsos et al., 2012). Through whole exome and sequencing of patients with congenital dyskeratosis (DC), the study found that there were compound heterozygous mutations (c.204 g > T and c.178-245del) in PARN. At the same time, B cells and NK blood cells were also detected to be reduced, the ratio of CD4:CD8 was inverted, and naive CD4 and CD8 cells were reduced (Zeng et al., 2020). However, in sepsis, lymphocytopenia and T-cell depletion are often found to be immunopathological characteristics (van der Poll et al., 2017). Therefore, the mechanism of PARN in sepsis deserves further discussion, especially whether PARN mediates immune cell apoptosis in sepsis deserves further attention.

In conclusion, we successfully performed molecular classification based on m7G-related genes in sepsis patients, and there were significant differences in prognosis among different subgroups. We also explored the key events such as signal pathways and immune infiltration within these subtypes. Our results can better explain the heterogeneity of sepsis patients and provide a basis for early intervention of sepsis patients. In addition, we identified two potential therapeutic targets for sepsis, namely NUDT4 and PARN, both of which are closely related to the prognosis of sepsis patients. Collectively, these findings will contribute to a better understanding of the occurrence and development of sepsis.

## Data Availability

The datasets presented in this study can be found in online repositories. The names of the repository/repositories and accession number(s) can be found in the article/supplementary material.

## References

[B1] Ali H. R. Chlon L. Pharoah P. D. Markowetz F. Caldas C. (2016). Patterns of immune infiltration in breast cancer and their clinical implications: A gene-expression-based retrospective study. PLoS Med. 13 (12), e1002194. 10.1371/journal.pmed.1002194 27959923 PMC5154505

[B2] Balatsos N. A. Maragozidis P. Anastasakis D. Stathopoulos C. (2012). Modulation of poly(A)-specific ribonuclease (PARN): Current knowledge and perspectives. Curr. Med. Chem. 19 (28), 4838–4849. 10.2174/092986712803341539 22834816

[B3] Barbie D. A. Tamayo P. Boehm J. S. Kim S. Y. Moody S. E. Dunn I. F. (2009). Systematic RNA interference reveals that oncogenic KRAS-driven cancers require TBK1. Nature 462 (7269), 108–112. 10.1038/nature08460 19847166 PMC2783335

[B4] Barrett T. Wilhite S. E. Ledoux P. Evangelista C. Kim I. F. Tomashevsky M. (2013). NCBI GEO: Archive for functional genomics data sets--update. Nucleic Acids Res. 41, D991–D995. 10.1093/nar/gks1193 23193258 PMC3531084

[B5] Barton R. Cerra F. B. (1989). The hypermetabolism. Multiple organ failure syndrome. Chest 96 (5), 1153–1160. 10.1378/chest.96.5.1153 2680321

[B6] Bermudes A. C. G. de Carvalho W. B. Zamberlan P. Muramoto G. Maranhão R. C. Delgado A. F. (2018). Changes in lipid metabolism in pediatric patients with severe sepsis and septic shock. Nutrition 47, 104–109. 10.1016/j.nut.2017.09.015 29429528

[B7] Bessman M. J. (2019). A cryptic activity in the Nudix hydrolase superfamily. Protein Sci. 28 (8), 1494–1500. 10.1002/pro.3666 31173659 PMC6635765

[B8] Binnie A. Tsang J. L. Y. Hu P. Carrasqueiro G. Castelo-Branco P. Dos Santos C. C. (2020). Epigenetics of sepsis. Crit. Care Med. 48 (5), 745–756. 10.1097/ccm.0000000000004247 32167492

[B9] Cao Q. Wang X. Jia L. Mondal A. K. Diallo A. Hawkins G. A. (2014). Inhibiting DNA Methylation by 5-Aza-2'-deoxycytidine ameliorates atherosclerosis through suppressing macrophage inflammation. Endocrinology 155 (12), 4925–4938. 10.1210/en.2014-1595 25251587 PMC4239421

[B10] Cavaillon J. M. Adib-Conquy M. (2005). Monocytes/macrophages and sepsis. Crit. Care Med. 33, S506–S509. 10.1097/01.ccm.0000185502.21012.37 16340435

[B11] Chen B. Khodadoust M. S. Liu C. L. Newman A. M. Alizadeh A. A. (2018). Profiling tumor infiltrating immune cells with CIBERSORT. Methods Mol. Biol. 1711, 243–259. 10.1007/978-1-4939-7493-1_12 29344893 PMC5895181

[B12] Chen J. Li K. Chen J. Wang X. Ling R. Cheng M. (2022). Aberrant translation regulated by METTL1/WDR4-mediated tRNA N7-methylguanosine modification drives head and neck squamous cell carcinoma progression. Cancer Commun. 42 (3), 223–244. 10.1002/cac2.12273 PMC8923133 35179319

[B13] Dahn M. S. Lange P. Lobdell K. Hans B. Jacobs L. A. Mitchell R. A. (1987). Splanchnic and total body oxygen consumption differences in septic and injured patients. Surgery 101 (1), 69–80. 3798330

[B14] Fleischmann C. Scherag A. Adhikari N. K. Hartog C. S. Tsaganos T. Schlattmann P. (2016). Assessment of global incidence and mortality of hospital-treated sepsis. Current estimates and limitations. Am. J. Respir. Crit. Care Med. 193 (3), 259–272. 10.1164/rccm.201504-0781OC 26414292

[B15] Gaieski D. F. Edwards J. M. Kallan M. J. Carr B. G. (2013). Benchmarking the incidence and mortality of severe sepsis in the United States. Crit. Care Med. 41 (5), 1167–1174. 10.1097/CCM.0b013e31827c09f8 23442987

[B16] Goel M. K. Khanna P. Kishore J. (2010). Understanding survival analysis: Kaplan-Meier estimate. Int. J. Ayurveda Res. 1 (4), 274–278. 10.4103/0974-7788.76794 21455458 PMC3059453

[B17] Grégoire C. Chasson L. Luci C. Tomasello E. Geissmann F. Vivier E. (2007). The trafficking of natural killer cells. Immunol. Rev. 220 (1), 169–182. 10.1111/j.1600-065X.2007.00563.x 17979846 PMC7165697

[B18] Huang M. Cai S. Su J. (2019). The pathogenesis of sepsis and potential therapeutic targets. Int. J. Mol. Sci. 20 (21), E5376. 10.3390/ijms20215376 PMC6862039 31671729

[B19] Huntington N. D. Vosshenrich C. A. Di Santo J. P. (2007). Developmental pathways that generate natural-killer-cell diversity in mice and humans. Nat. Rev. Immunol. 7 (9), 703–714. 10.1038/nri2154 17717540

[B20] Hussell T. Bell T. J. (2014). Alveolar macrophages: Plasticity in a tissue-specific context. Nat. Rev. Immunol. 14 (2), 81–93. 10.1038/nri3600 24445666

[B21] Iwashyna T. J. Cooke C. R. Wunsch H. Kahn J. M. (2012). Population burden of long-term survivorship after severe sepsis in older Americans. J. Am. Geriatr. Soc. 60 (6), 1070–1077. 10.1111/j.1532-5415.2012.03989.x 22642542 PMC3374893

[B22] Jairath N. K. Farha M. W. Srinivasan S. Jairath R. Green M. D. Dess R. T. (2020). Tumor immune microenvironment clusters in localized prostate adenocarcinoma: Prognostic impact of macrophage enriched/plasma cell non-enriched subtypes. J. Clin. Med. 9 (6), E1973. 10.3390/jcm9061973 PMC7356642 32599760

[B23] Kotas M. E. Matthay M. A. (2018). Mesenchymal stromal cells and macrophages in sepsis: New insights. Eur. Respir. J. 51 (4), 1800510. 10.1183/13993003.00510-2018 29700107

[B24] Lang C. H. Frost R. A. Vary T. C. (2007). Regulation of muscle protein synthesis during sepsis and inflammation. Am. J. Physiol. Endocrinol. Metab. 293 (2), E453–E459. 10.1152/ajpendo.00204.2007 17505052

[B25] Langfelder P. Horvath S. (2008). Wgcna: an R package for weighted correlation network analysis. BMC Bioinforma. 9, 559. 10.1186/1471-2105-9-559 PMC2631488 19114008

[B26] Li X. Y. Wang S. L. Chen D. H. Liu H. You J. X. Su L. X. (2022). Construction and validation of a m7G-related gene-based prognostic model for gastric cancer. Front. Oncol. 12, 861412. 10.3389/fonc.2022.861412 35847903 PMC9281447

[B27] Liao M. Liu Y. Yuan J. Wen Y. Xu G. Zhao J. (2020). Single-cell landscape of bronchoalveolar immune cells in patients with COVID-19. Nat. Med. 26 (6), 842–844. 10.1038/s41591-020-0901-9 32398875

[B28] Liberzon A. Birger C. Thorvaldsdóttir H. Ghandi M. Mesirov J. P. Tamayo P. (2015). The Molecular Signatures Database (MSigDB) hallmark gene set collection. Cell Syst. 1 (6), 417–425. 10.1016/j.cels.2015.12.004 26771021 PMC4707969

[B29] Liu N. Zhou K. I. Parisien M. Dai Q. Diatchenko L. Pan T. (2017a). N6-methyladenosine alters RNA structure to regulate binding of a low-complexity protein. Nucleic Acids Res. 45 (10), 6051–6063. 10.1093/nar/gkx141 28334903 PMC5449601

[B30] Liu X. Wu S. Yang Y. Zhao M. Zhu G. Hou Z. (2017b). The prognostic landscape of tumor-infiltrating immune cell and immunomodulators in lung cancer. Biomed. Pharmacother. 95, 55–61. 10.1016/j.biopha.2017.08.003 28826097

[B31] Luo Y. Yao Y. Wu P. Zi X. Sun N. He J. (2022). The potential role of N(7)-methylguanosine (m7G) in cancer. J. Hematol. Oncol. 15 (1), 63. 10.1186/s13045-022-01285-5 35590385 PMC9118743

[B32] Malbec L. Zhang T. Chen Y. S. Zhang Y. Sun B. F. Shi B. Y. (2019). Dynamic methylome of internal mRNA N(7)-methylguanosine and its regulatory role in translation. Cell Res. 29 (11), 927–941. 10.1038/s41422-019-0230-z 31520064 PMC6889513

[B33] Mildvan A. S. Xia Z. Azurmendi H. F. Saraswat V. Legler P. M. Massiah M. A. (2005). Structures and mechanisms of Nudix hydrolases. Arch. Biochem. Biophys. 433 (1), 129–143. 10.1016/j.abb.2004.08.017 15581572

[B34] Murch O. Collin M. Hinds C. J. Thiemermann C. (2007). Lipoproteins in inflammation and sepsis. I. Basic science. Intensive Care Med. 33 (1), 13–24. 10.1007/s00134-006-0432-y 17093985

[B35] Purcarea A. Sovaila S. (2020). Sepsis, a 2020 review for the internist. Rom. J. Intern Med. 58 (3), 129–137. 10.2478/rjim-2020-0012 32396142

[B36] Ralainirina N. Poli A. Michel T. Poos L. Andrès E. Hentges F. (2007). Control of NK cell functions by CD4+CD25+ regulatory T cells. J. Leukoc. Biol. 81 (1), 144–153. 10.1189/jlb.0606409 16959895

[B37] Reisinger A. C. Posch F. Hackl G. Marsche G. Sourij H. Bourgeois B. (2021). Branched-chain amino acids can predict mortality in ICU sepsis patients. Nutrients 13 (9), 3106. 10.3390/nu13093106 34578983 PMC8469152

[B38] Remick D. G. (2007). Pathophysiology of sepsis. Am. J. Pathol. 170 (5), 1435–1444. 10.2353/ajpath.2007.060872 17456750 PMC1854939

[B39] Rocheteau P. Chatre L. Briand D. Mebarki M. Jouvion G. Bardon J. (2015). Sepsis induces long-term metabolic and mitochondrial muscle stem cell dysfunction amenable by mesenchymal stem cell therapy. Nat. Commun. 6, 10145. 10.1038/ncomms10145 26666572 PMC4682118

[B40] Roundtree I. A. Evans M. E. Pan T. He C. (2017). Dynamic RNA modifications in gene expression regulation. Cell 169 (7), 1187–1200. 10.1016/j.cell.2017.05.045 28622506 PMC5657247

[B41] Scott M. J. Hoth J. J. Turina M. Woods D. R. Cheadle W. G. (2006). Interleukin-10 suppresses natural killer cell but not natural killer T cell activation during bacterial infection. Cytokine 33 (2), 79–86. 10.1016/j.cyto.2005.12.002 16488621

[B42] Shih C. C. Liao M. H. Hsiao T. S. Hii H. P. Shen C. H. Chen S. J. (2016). Procainamide inhibits DNA methylation and alleviates multiple organ dysfunction in rats with endotoxic shock. PLoS One 11 (9), e0163690. 10.1371/journal.pone.0163690 27661616 PMC5035080

[B43] Singer M. Deutschman C. S. Seymour C. W. Shankar-Hari M. Annane D. Bauer M. (2016). The third international consensus definitions for sepsis and septic shock (Sepsis-3). Jama 315 (8), 801–810. 10.1001/jama.2016.0287 26903338 PMC4968574

[B44] Swift S. Tucker A. Vinciotti V. Martin N. Orengo C. Liu X. (2004). Consensus clustering and functional interpretation of gene-expression data. Genome Biol. 5 (11), R94. 10.1186/gb-2004-5-11-r94 15535870 PMC545785

[B45] van der Poll T. van de Veerdonk F. L. Scicluna B. P. Netea M. G. (2017). The immunopathology of sepsis and potential therapeutic targets. Nat. Rev. Immunol. 17 (7), 407–420. 10.1038/nri.2017.36 28436424

[B46] Xiao F. Guo Z. Yan Q. (2015). The metabolic change in citrulline and its use in sepsis. Zhonghua Wei Zhong Bing Ji Jiu Yi Xue 27 (6), 534–537. 10.3760/cma.j.issn.2095-4352.2015.06.024 26049199

[B47] Zeng T. Lv G. Chen X. Yang L. Zhou L. Dou Y. (2020). CD8(+) T-cell senescence and skewed lymphocyte subsets in young Dyskeratosis Congenita patients with PARN and DKC1 mutations. J. Clin. Lab. Anal. 34 (9), e23375. 10.1002/jcla.23375 32452087 PMC7521304

[B48] Zhang B. Li D. Wang R. (2022). Transcriptome profiling of N7-methylguanosine modification of messenger RNA in drug-resistant acute myeloid leukemia. Front. Oncol. 12, 926296. 10.3389/fonc.2022.926296 35865472 PMC9294171

[B49] Zhang Y. Y. Ning B. T. (2021). Signaling pathways and intervention therapies in sepsis. Signal Transduct. Target. Ther. 6 (1), 407. 10.1038/s41392-021-00816-9 34824200 PMC8613465

